# Structural basis for polyuridine tract recognition by SARS-CoV-2 Nsp15

**DOI:** 10.1093/procel/pwae009

**Published:** 2024-04-13

**Authors:** Fumiaki Ito, Hanjing Yang, Z Hong Zhou, Xiaojiang S Chen

**Affiliations:** Molecular and Computational Biology, Department of Biological Sciences, University of Southern California, Los Angeles, CA 90089, United States; Department of Microbiology, Immunology and Molecular Genetics, University of California, Los Angeles, CA 90095, United States; California NanoSystems Institute, University of California, Los Angeles, CA90095, United States; Molecular and Computational Biology, Department of Biological Sciences, University of Southern California, Los Angeles, CA 90089, United States; Department of Microbiology, Immunology and Molecular Genetics, University of California, Los Angeles, CA 90095, United States; California NanoSystems Institute, University of California, Los Angeles, CA90095, United States; Molecular and Computational Biology, Department of Biological Sciences, University of Southern California, Los Angeles, CA 90089, United States; Genetic, Molecular and Cellular Biology Program, Keck School of Medicine, University of Southern California, Los Angeles, CA 90089, United States; Norris Comprehensive Cancer Center, University of Southern California, Los Angeles, CA 90089, United States; Center of Excellence in NanoBiophysics, University of Southern California, Los Angeles, CA 90089, United States

## Dear Editor,

Coronaviruses have evolved a wide array of tactics to evade host antiviral immunity. When host cells detect invading foreign nucleic acids including viral genome and viral replication intermediates, they activate interferon (IFN) signaling via cytoplasmic pattern recognition receptors (Kang et al., 2002; Kato et al., 2006). Remarkable stealth activities exhibited by coronaviruses are facilitated by a series of non-structural proteins (Nsps), such as Nsp15 (Deng and Baker, 2018; Deng et al., 2019; Hackbart et al., 2020), which is a uridine-specific endoribonuclease that mediates evasion of host detection of viral double-stranded RNA (dsRNA) (Bhardwaj et al., 2004; Deng et al., 2017; Frazier et al., 2021; Ivanov et al., 2004). Nsp15 targets a polyuridine [poly(U)] tract on the coronavirus negative strand RNA (Hackbart et al., 2020). Nsp15 limits the abundance and length of poly(U) within the negative strand RNA 5ʹ-extension by trimming down the initially synthesized poly(U) lead sequence to the optimal length that can suppress dsRNA formation but still serves as a template for poly(A) tail of the positive strand genome of the coronaviruses (Hofmann and Brian, 1991). Prior Nsp15 structures have provided some insights into its binding to both ssRNA and dsRNA (Frazier et al., 2021, 2022; Kim et al., 2020), but the precise molecular mechanisms underlying the recognition of poly(U) by Nsp15 remain incomplete. Here we reconstituted a complex of SARS-CoV-2 Nsp15 with a viral replicative dsRNA intermediate containing 3ʹ-end of the viral genome followed by a 20-bp poly(A/U) extension. Cryogenic electron microscopy (cryoEM) revealed Nsp15 hexamer structures at 2.3–3.3 Å resolution at various functional states, including RNA-free and two dsRNA-bound states. Comparison of these structures shows that the poly(U) tract of the sequence is recognized by an Nsp15 hexamer via direct contact with three subunits in two distinct states. The target uracil is dislodged from the base-pairing of the dsRNA by amino acid residues W332 and M330 of Nsp15, and the dislodged base is entrapped at the endonuclease active site center. Thus, the active site utilizes a base-flipping mechanism to hold the target uracil base in the endonuclease catalytic center for cleavage. Up to 20 A/U base pairs are anchored on the Nsp15 hexamer, which explains the basis for a substantially shortened poly(U) sequence in the negative strand coronavirus genome compared to the long poly(A) tail in its positive strand. Overall, our structures reveal how Nsp15 binds to the poly(A/U) sequence of its genomic replicative dsRNA intermediate to evade host antiviral response.

**Figure 1.**
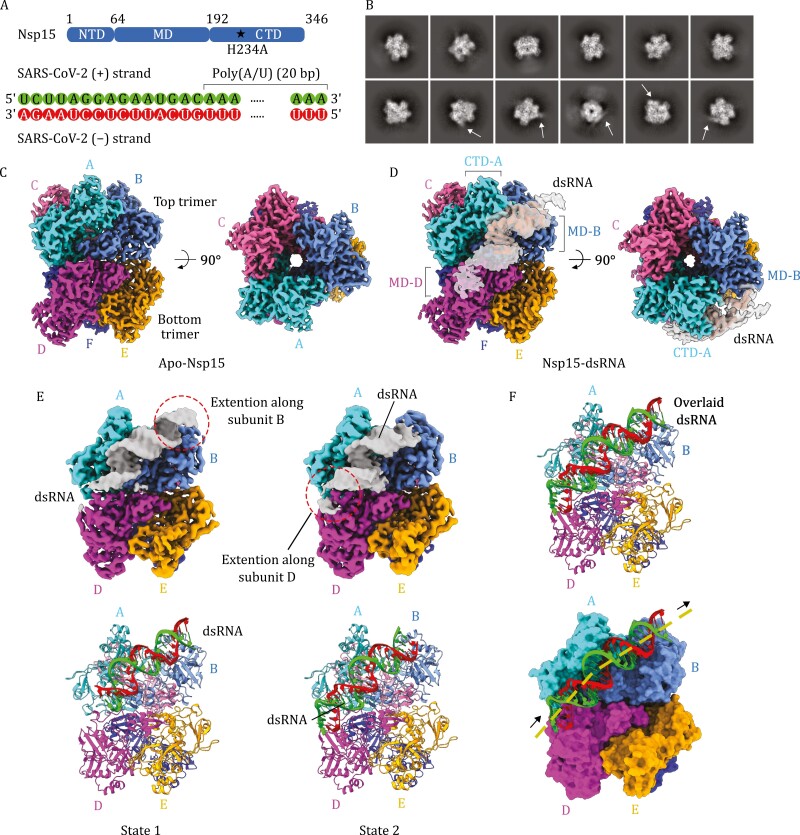
CryoEM reconstructions of apo- and two states of RNA-bound SARS-CoV-2 Nsp15. (A) Schematic representation of the domain organization and construct design of Nsp15 (top). dsRNA substrate sequence used for the reconstitution of Nsp15-RNA complex (bottom). (B) 2D class averages of the reconstituted Nsp15-RNA complex. A subset of the 2D classes shows extra density stemming from the core of the 2D density (indicated by arrows). (C) Two orthogonal views of the cryoEM reconstruction of apo-Nsp15 at 2.3 Å resolution. (D) Two orthogonal views of the cryoEM reconstruction of the Nsp15-RNA complex at 2.7 Å resolution. RNA density is shown in two different isosurface threshold levels to show both high-resolution and low-resolution features. (E) CryoEM density maps (top) and the corresponding atomic models (bottom) of the Nsp15-RNA state 1 (left) and state 2 (right) structures. The RNA chains in green and red correspond to positive and negative strands of the coronavirus genome, respectively. (F) Overlay of the RNA models from the two states on the consensus Nsp15 structure in the ribbon model (top) and surface model (bottom). The dotted line indicates the trajectory of the bound dsRNA.

To understand the mechanism of poly(U) targeting by SARS-CoV-2 Nsp15, we reconstituted a ribonucleoprotein complex of Nsp15 and a 35-bp dsRNA substrate comprising the final 15-bp of 3ʹ-end of the SARS-CoV-2 genome and 20-bp poly(A/U) extension, which represents a coronavirus genome replication intermediate. Nsp15 C-terminal domain (CTD) belongs to a family of endoribonuclease with two conserved catalytic histidine residues (H234 and H249), which serve as general acid and general base, respectively, to attack the 3ʹ-phosphate of the target uridine (Kim et al., 2020). To capture the RNA substrate bound to Nsp15 and trap the target uracil base at the nuclease active site, catalytically inactive mutant H234A was used for the reconstitution (Fig. 1A). SARS-CoV-2 Nsp8, a previously hypothesized cofactor of the Nsp15, was also added to facilitate the Nsp15-RNA interaction (Athmer et al., 2017; Zhang et al., 2018). CryoEM imaging shows that an equimolar ratio of Nsp15:RNA resulted in mostly apo-Nsp15 (Figs. S1 and S2; and data set 1 in Fig. S2) and that an excess amount of RNA with the Nsp15:RNA ratio of 1:10 yielded a complex with the available RNA binding sites within the functional oligomer of Nsp15 occupied by RNA (data sets 2 and 3 in Fig. S2).

Two-dimensional classification of the reconstituted Nsp15-RNA complex particles in data sets 2 and 3 showed that a subset of 2D class averages has extra helical densities stemming from the core of the 2D densities (Fig. 1B). Multiclass *ab initio* 3D reconstruction showed that 37% of the particles belong to RNA-free apo-Nsp15, and 39% belong to Nsp15 in complex with obvious dsRNA (Fig. S2). The apo-Nsp15 was reconstructed as a homohexamer with D3 symmetry and refined to 2.3 Å resolution (Table S1). The hexamer forms a barrel-like architecture with a central channel, which consists of a head-to-head stack of two trimers (defined as top and bottom) (Figs. 1C, S3 and S4). The RNA-bound form was reconstructed as a homohexamer with clear A-form-like RNA duplex density and refined to 2.7 Å resolution (Table S1). dsRNA density is diagonally attached to the outer periphery of the hexameric barrel and occupies a shallow groove between two subunits of the top trimer (defined as subunits A and B) (Figs. 1D, S5, and S6). The nuclease active site centers are located near the subunit interface between the neighboring subunits within the top or bottom trimers and the bound dsRNA fully shields the active site of the subunit A. One end of the dsRNA stretches along a groove between the CTD of subunit A and the middle domain (MD) of subunit B, extending upwards beyond the top trimer. The other end of the dsRNA stretches towards the MD of the subunit D within the bottom trimer. No extra densities were observed in the central channel of the hexamer. Although we added an excess quantity of the substrate RNA (10-fold of Nsp15 in molarity), we only observed Nsp15 hexamer with a single piece of dsRNA as a substrate-bound form. The active sites of the other five subunits are unoccupied. Extensive 3D classification did not yield any 3D classes containing more than one dsRNA piece per Nsp15 hexamer. These observations likely indicate that Nsp15 hexamer is compatible with only one dsRNA substrate at a time. Of note, the density for the hypothetical cofactor Nsp8 was not observed in any of the 2D or 3D classes we obtained, likely due to the weak interaction between Nsp15 and Nsp8.

We noticed that both ends of the bound dsRNA had relatively weak density (Fig. 1D). We, therefore, hypothesized that there is conformational or compositional variability in the bound RNA structure and that the observed RNA-bound form could be an average of multiple different states. Further heterogeneous 3D refinement revealed that the dsRNA-bound form can be subclassified into two states: A class with dsRNA extending towards subunit B in the upper trimer (state 1) and the other class with dsRNA extending towards subunit D in the bottom trimer (state 2) (Figs. 1E and Fig. S7). In the state 1 structure, the full 20 A/U pairs are readily visible, including 11-bp preceding the target U at the active site (defined as U_0_) and 9-bp following the U_0_, resulting in the U_0_ being located at the 12th base from 3ʹ-end of the SARS-CoV-2 genome. The state 2 structure also showed the full 20 A/U pairs, including 3-bp preceding the U_0_ and 16-bp following the U_0_, resulting in the U_0_ being located at the 4th base from 3ʹ-end of the SARS-CoV-2 genome. Outside the poly(A/U) tract, the state 1 and 2 structures had extra 3- and 7-bp, respectively, corresponding to the 3ʹ-end of the SARS-CoV-2 genome. The two states of RNA-bound form likely indicate that Nsp15 can target a range of uridines for cleavage, and the 4th and 12th uridines from the 3ʹ-end within the poly(U) may potentially be a preferred sites for degradation.

**Figure 2.**
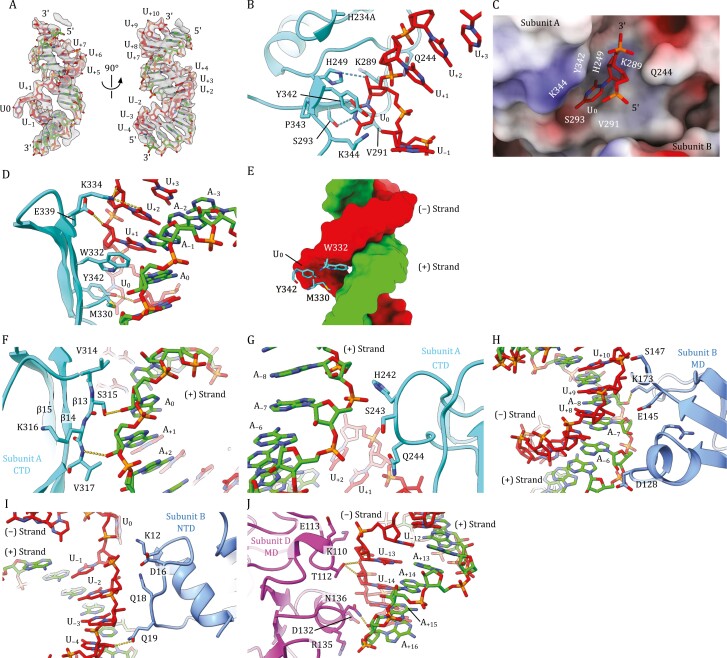
Uracil base flipping at nuclease active site center and overall dsRNA interactions with Nsp15 hexamer across three subunits. (A) CryoEM density and the corresponding atomic model of the RNA bound to Nsp15. Two orthogonal views of the bound dsRNA density and its model are shown. (B) Structure of the endonuclease active site center and recognition mechanism of flipped scissile U_0_ base. (C) Surface electrostatic potential of the Nsp15 around the endonuclease active site center and the interactions with the target U_0_ with both 3ʹ- and 5ʹ-phosphates (depicted in sticks). The surface area is colored according to the calculated electrostatic potential from −10.0 kT/e to +10.0 kT/e. (D) Recognition of the open major groove of the dsRNA substrate by Nsp15. W332 is intercalated into the space that would have been occupied by the flipped U_0_ base within the RNA duplex. M330 and Y342 additionally create the hydrophobic surface to facilitate the major groove interaction. Two polar residues K334 and E339 interact with U_+2_ and U_+1_, respectively. (E) The key hydrophobic residues responsible for base-flipping. The side chain of W332 is deeply intercalated into the major groove of the RNA duplex. M330 and Y342 additionally participate in the major groove interaction. (F) Interface between a patch of −^314^VSKV^317^− of subunit A’s CTD and A_0_ and A_+1_ of the positive strand. (G) Interface between a patch of −^242^HSQ^244^− of subunit A’s CTD and A_−8_ and A_−7_ of the positive strand and U_+1_ of the negative strand. (H) Interface between subunit B’s MD and U_+10_ of the negative strand and A_−8_, A_−6_ of the positive strand. (I) Interface between subunit B’s NTD and the U_−4_–U_−1_ of the negative strand. (J) Interface between subunit D’s MD and U_−14_–U_−12_ of the negative strand and A_+16_ of the positive strand. The consensus form of the Nsp15-RNA structure is displayed in (B–J).

The dsRNA is firmly held by the Nsp15 hexamer with a slight bent near the groove between the CTD of subunit A and the NTD of subunit B (Fig. 1F). Within the central region of the dsRNA, we observed unpaired bases: one base is flipped outside from the RNA duplex while its complementary counterpart remains orienting inwards within the duplex (Fig. 2A). U/A pair was modeled at this location and the flipped uracil is designated as U_0_. The local resolution of the bound RNA ranges between 2.4 Å and 4.0 Å with the highest resolution around the flipped U_0_ base (Fig. S5). A total of 17 A/U base pairs were confidently built for the consensus Nsp15-RNA structure including the 10-bp preceding the flipped U_0_ and 6-bp following the U_0_. Outside this patch, the base density features are insufficient to distinguish their identity.

At the endonuclease active site pocket, the pyrimidine ring of the flipped uracil base is sandwiched between two hydrophobic residues Y342 and V291. The aliphatic chain of K344 additionally constitutes this hydrophobic pocket to hold the uracil base. S293 plays a key role in conferring the selectivity for the target uracil as a hydroxyl of the S293 side chain recognizes the N3 atom of the uracil base to form a hydrogen bond. The main chain nitrogen of S293 forms another hydrogen bond with the O_2_ atom of the uracil base. The catalytic H249 is located near the scissile 3ʹ-phosphate of U_0_ and its imidazole ring forms a hydrogen bond with the 2ʹ-OH of the ribose ring of U_0_. As expected, the inactivated catalytic histidine (H234A) is located near H249 and the scissile 3ʹ-phosphate. Two polar residues Q244 and K289 additionally surround the 3ʹ-phosphate, thereby stabilizing the position of the target uridine (Fig. 2B and 2C).

The space that would have been occupied by the U_0_ base within the RNA duplex is partially occupied by W332 from the three-stranded anti-parallel β-sheet of CTD. W332 together with M330 and Y342 from the same β-sheet creates a hydrophobic surface and intercalates into the open major groove at this location (Fig. 2D and 2E). Notably, W332 positions itself directly across the orphan A_0_ base and stabilizes the adjacent U_+1_ base by forming a stacking interaction. These three residues, M330, W332, and Y342, responsible for dislodging the target uracil base are completely conserved across coronaviruses, highlighting their importance (Fig. S8). Two polar residues K334 and E339 near the top edge of the β-sheet interact with the U_+2_ and U_+1_ in the negative strand, respectively. A primary amine of K334 forms hydrogen bonds with the O2 atom of the U_+2_ base, and 2ʹ-OH of the ribose ring of U_+2_. A side chain carboxyl of E339 forms hydrogen bonds with 2ʹ-OH of the ribose ring of U_+1_ (Fig. 2D and 2E).

The dsRNA substrate contacts three subunits (subunits A, B, and D) extensively on the sidewall of the Nsp15 hexameric barrel (Fig. 1F). Both the negative and positive strands make substantial contacts with Nsp15 across approximately two and a half turns of the double-stranded helix. Aside from the base pair that involves flipped U_0_, base pairing is well-maintained throughout the duplex. Outside the endonuclease active site pocket anchoring the flipped U_0_ base, subunit A has two additional interface areas in either direction of the active site (Fig. 2F and 2G). The first interface area includes the first strand of the three-stranded β-sheet in the CTD. A patch of −^314^VSKV^317^− in β13 is in close contact with the orphan nucleotide A_0_ and A_+1_ in the positive-strand RNA. S315 forms a hydrogen bond with the 2ʹ-hydroxyl group of the ribose ring of the A_0_, while the backbone nitrogen of V317 forms another hydrogen bond with the 2ʹ-hydroxyl of the ribose ring of A_+1_. The second interface in subunit A is near a patch of −^242^HSQ^244^− within a loop region, which supports the backbone of the positive strand between positions A_−8_ to A_−6_. Subunit B, on the other hand, interacts with the RNA through its MD and NTD (Fig. 2H and 2I). The surface area comprised of hydrophilic residues S128, E145, S147, and K173 in subunit B’s MD contacts the major groove near U_+8_ to U_+10_ in the negative strand and A_−8_ to A_−6_ in the positive strand (Fig. 2H). Another polar surface comprised of residues K12, D16, Q18, and Q19 in subunit B’s NTD contacts the backbone of the negative strand near U_−4_ to U_−1_ (Fig. 2I). Lastly, Nsp15-RNA state 2 structure has an additional interface in subunit D from the bottom trimer. A hydrophilic surface comprised of the polar residues K110, T112, E113, D132, N136, and R135 in subunit D’s MD contacts the minor groove area near U_−14_, and U_−13_ in the negative strand and A_+15_, and A_+16_ in the positive strand, further stabilizing the bound dsRNA (Fig. 2J).

Comparison of subunit A structures in its RNA-bound form and its apo-form showed an r.m.s.d. of 0.388 Å, indicating that the binding of RNA does not induce significant global conformational changes to Nsp15 protomer. Yet, the high-resolution structures of both apo- and RNA-bound forms of Nsp15 allowed us to identify notable local structural changes. First, upon binding of RNA, there is a subtle linear shift of β-strands 14 and 15, accompanied by their connecting β-turn (−^334^KDGH^337^−), towards the bound dsRNA. This shift is likely caused by the presence of W332 on the β-strand 14 (Fig. S9A). Second, the C-terminal tail of the subunit A including the terminal residues −^344^KLQ^346^ underwent a structural remodeling upon RNA binding. Superimposition of the apo-Nsp15 and RNA-bound subunit A structures showed that the C-terminal end glutamine residue clashes with the flipped uracil base. In the RNA-bound structure, the ^344^KLQ^346^ patch swung away from the active site pocket, allowing the substrate uracil base to fit into the pocket (Fig. S9B). The rest of the subunits (B to F) did not display any noticeable structural changes upon RNA binding. Interestingly, the cryoEM density for residues W332 and M330 in subunit A, which play key roles in the base-flipping of the target uracil, are more clearly defined than the same residues in the other subunits, indicating that the side chains of these residues are stabilized by the RNA binding (Fig. S9C).

Prior crystallographic and cryoEM studies of SARS-CoV-2 Nsp15 have provided insights into its binding to both ssRNA and dsRNA (Frazier et al., 2021, 2022; Kim et al., 2021). The target uracil base recognition mode observed in our structure is consistent with both short ssRNA-bound and dsRNA-bound structures (Fig. S10) (Frazier et al., 2022; Kim et al., 2021). The overall dsRNA binding mode observed in our Nsp15-RNA structures resembles the structure of Nsp15 bound to a 52-bp dsRNA that was adopted from a substrate of the Drosophila Dicer-2 (Frazier et al., 2022). Notably, the structure with 52-bp dsRNA shows the adenine at the +1 position of the target strand. Our high-resolution structure shows that uridine can also be readily accommodated at this location. Interestingly, the comparison of the structures around the target U_0_ base revealed that the position of W332 is finely adjusted for promoting the stacking interaction with the U_+1_ base while the flipped U_0_ base remains precisely at the same position (Fig. S10).

In summary, our structures of the Nsp15-RNA complex in two states reveal direct interactions between Nsp15 and poly(A/U) RNA, its only known physiological substrate. These structures offer snapshots that inform how SARS-CoV-2 camouflages itself in infected cells to escape the host detection of viral RNA. Given the high sequence conservation of endoribonuclease among known coronavirus lineages and SARS-CoV-2 variants known to date, targeting Nsp15 activity may be a promising therapeutic strategy against the current and future SARS-CoV-2 variants. Inhibitors of the Nsp15 activity would preserve the natural innate immune responses against dsRNA derived from the coronavirus genome, giving rise to broad-spectrum anti-viral drugs.

## Supplementary information

The online version contains supplementary material available at https://doi.org/10.1093/procel/pwae009.

pwae009_suppl_Supplementary_Materials

## Footnotes

Electron microscopy data were collected at the Core Center of Excellence in Nano Imaging (CNI) at USC. CryoEM data was computed at Center for Advanced Research Computing (CARC) at USC. We thank Htet Khant, Carolyn Marks, and John Curulli for assisting with the operation and maintenance of transmission electron microscopes at CNI, Tomek Osinski for assisting with computing work at CARC, and Cornelius Gati for advice on cryo-EM sample preparation and data processing. The atomic models have been deposited in the PDB with accession codes: 8UD2 (apo-Nsp15), 8UD3 (Nsp15-RNA consensus form), 8UD4 (Nsp15-RNA state 1), and 8UD5 (Nsp15-RNA state 2). The cryoEM maps have been deposited in the EMDB with accession codes: EMD-42144 (apo-Nsp15), EMD-42145 (Nsp15-RNA consensus form), EMD-42146 (Nsp15-RNA state 1), and EMD-42147 (Nsp15-RNA state 2). Raw electron microscopy data files have been deposited in the Electron Microscopy Public Image Archive (EMPIAR) with accession code EMPIAR-11880. This work was supported in part by grants R01AI150524 to X.S.C. and R01GM071940 to Z.H.Z. from the National Institutes of Health.

The authors declare no competing financial interests.

All authors reviewed and approved the paper. X.S.C. and Z.H.Z. supervised the project and acquired the funding. F.I. conceived the project and designed the experiments. F.I. and H.Y. generated clones and purified the proteins. F.I. performed the CryoEM grid screening, data collection, image processing, and atomic model building. F.I. wrote the manuscript with inputs from all authors.

## References

[CIT0001] Athmer J , Fehr AR, Grunewald M et al. In situ tagged Nsp15 reveals interactions with coronavirus replication/transcription complex-associated proteins. Mbio 2017;8:e02320–e02316. 28143984 10.1128/mBio.02320-16 PMC5285509

[CIT0002] Bhardwaj K , Guarino L, Kao CC. The severe acute respiratory syndrome coronavirus Nsp15 protein is an endoribonuclease that prefers manganese as a cofactor. J Virol 2004;78:12218–12224. 15507608 10.1128/JVI.78.22.12218-12224.2004 PMC525082

[CIT0003] Deng X , Baker SC. An “Old” protein with a new story: coronavirus endoribonuclease is important for evading host antiviral defenses. Virology 2018;517:157–163. 29307596 10.1016/j.virol.2017.12.024 PMC5869138

[CIT0004] Deng X , Hackbart M, Mettelman RC et al. Coronavirus nonstructural protein 15 mediates evasion of dsRNA sensors and limits apoptosis in macrophages. Proc Natl Acad Sci U S A 2017;114:E4251–E4260. 28484023 10.1073/pnas.1618310114 PMC5448190

[CIT0005] Deng X , Van Geelen A, Buckley AC et al. Coronavirus endoribonuclease activity in porcine epidemic diarrhea virus suppresses Type I And Type III interferon responses. J Virol 2019;93. 10.1128/JVI.02000-18 PMC6450110 30728254

[CIT0006] Frazier MN , Dillard LB, Krahn JM et al. Characterization Of SARS2 Nsp15 nuclease activity reveals it’s mad about U. Nucleic Acids Res 2021;49:10136–10149. 34403466 10.1093/nar/gkab719 PMC8385992

[CIT0007] Frazier MN , Wilson IM, Krahn JM et al. Flipped over U: structural basis for dsRNA cleavage by the SARS-Cov-2 endoribonuclease. Nucleic Acids Res 2022;50:8290–8301. 35801916 10.1093/nar/gkac589 PMC9371922

[CIT0008] Hackbart M , Deng X, Baker SC. Coronavirus endoribonuclease targets viral polyuridine sequences to evade activating host sensors. Proc Natl Acad Sci U S A 2020;117:8094–8103. 32198201 10.1073/pnas.1921485117 PMC7149396

[CIT0009] Hofmann MA , Brian DA. The 5’ end of coronavirus minus-strand RNAs contains a short poly(U) tract. J Virol 1991;65:6331–6333. 1920635 10.1128/jvi.65.11.6331-6333.1991 PMC250348

[CIT0010] Ivanov KA , Hertzig T, Rozanov M et al. Major genetic marker of nidoviruses encodes a replicative endoribonuclease. Proc Natl Acad Sci U S A 2004;101:12694–12699. 15304651 10.1073/pnas.0403127101 PMC514660

[CIT0011] Kang DC , Gopalkrishnan RV, Wu Q et al. mda-5: an interferon-inducible putative RNA helicase with double-stranded RNA-dependent ATPase activity and melanoma growth-suppressive properties. Proc Natl Acad Sci U S A 2002;99:637–642. 11805321 10.1073/pnas.022637199 PMC117358

[CIT0012] Kato H , Takeuchi O, Sato S et al. Differential roles of MDA5 and RIG-I helicases in the recognition of RNA viruses. Nature 2006;441:101–105. 16625202 10.1038/nature04734

[CIT0013] Kim Y , Jedrzejczak R, Maltseva NI et al. Crystal structure Of Nsp15 Endoribonuclease NendoU from SARS-CoV-2. Protein Sci 2020;29:1596–1605. 32304108 10.1002/pro.3873 PMC7264519

[CIT0014] Kim Y , Wower J, Maltseva N et al. Tipiracil binds to uridine site and inhibits Nsp15 Endoribonuclease NendoU from SARS-Cov-2. Commun Biol 2021;4:193. 33564093 10.1038/s42003-021-01735-9 PMC7873276

[CIT0015] Zhang L , Li L, Yan L et al. Structural And biochemical characterization of endoribonuclease Nsp15 Encoded By Middle East respiratory syndrome coronavirus. J Virol 2018;92. 10.1128/JVI.00893-18 PMC6206473 30135128

