# Supplementary material for: Structural basis for polyuridine tract recognition by SARS-CoV-2 Nsp15
Source: Protein Cell. 2024 Apr 13;15(7):547–52. doi: 10.1093/procel/pwae009 (PMC11214832; doi:10.1093/procel/pwae009)
Supplement: pwae009_suppl_Supplementary_Materials [file pwae009_suppl_supplementary_materials.pdf]

Supplemental Information

Title: Structural basis for polyuridine tract recognition by SARS-CoV-2 Nsp15

Fumiaki Ito[1,2,3], Hanjing Yang[1], Z. Hong Zhou[2,3], and Xiaojiang S. Chen[1,4,5,6*]

[1]Molecular and Computational Biology, Department of Biological Sciences, University of Southern California, Los Angeles, CA 90089, USA

[2]Department of Microbiology, Immunology and Molecular Genetics, University of California, Los Angeles, CA90095, USA

[3]California NanoSystems Institute, University of California, Los Angeles, CA90095, USA

[4]Genetic, Molecular and Cellular Biology Program, Keck School of Medicine, [5]Norris Comprehensive Cancer Center, and [6]Center of Excellence in NanoBiophysics, University of Southern California, Los Angeles, CA90089, USA

*To whom correspondence should be addressed

Phone: Tel: +1(213)740-5487; FAX: +1(213)740-4340; Email: xiaojiac@usc.edu

Materials and methods

Plasmids

Nsp15 from SARS-CoV-2 isolate WA-CDC-02982586-001/2020 (GenBank: MN985325.1, residues 1-346) with His_6-tag at N-terminus was cloned into Champion™ pET SUMO vector by excluding SUMO fusion tag, and Nsp8 from the same SARS-CoV-2 isolate (residues 1-198) was cloned into pET28a vector with His_6-tag at N-terminus. Cloning and mutagenesis were performed with In-Fusion cloning and PrimeSTAR mutagenesis (Clontech) by following the manufacturer's instructions. The sequences of all the constructs were verified by Sanger DNA sequencing (Azenta Life Sciences). The multiple sequence alignments were generated with Linnaeo (https://github.com/beowulfey/linnaeo).

Protein expression and purification

His_6-Nsp15 catalytically inactive mutant H234A and wild-type His_6-Nsp8 expression vectors were transformed into the *E. coli* strains BL21(DE3). The *E. coli* cells harboring the expression vectors were grown in LB medium at 37°C until the OD_{600} reaches 0.6. The recombinant proteins were induced by 0.2 mM isopropyl β-D-1-thiogalactopyranoside (IPTG) at 16°C for 18 hours.

For Nsp15, the cell pellets were resuspended with the buffer (25 mM HEPES-NaOH (pH 7.5), 500 mM NaCl, and 0.5 mM TCEP) containing RNase A (0.1 mg/ml, Qiagen), lysed by sonication, and cellular debris was removed by centrifugation. The supernatant containing the His_6-Nsp15 was loaded onto the Ni-NTA agarose column (Qiagen). The nickel column was extensively washed with wash buffer (25 mM HEPES-NaOH (pH 7.5), 500 mM NaCl, 50 mM imidazole, and 0.5 mM TCEP) and the protein was eluted with elution buffer (25 mM HEPES-NaOH (pH 7.5), 500 mM NaCl, 500 mM imidazole, and 0.5 mM TCEP). The eluted proteins were concentrated and subjected to Superdex 200 Increase 10/300 GL column (Cytiva) equilibrated with the buffer (25 mM HEPES-NaOH (pH 7.5), 150 mM NaCl, and 0.5 mM TCEP). The peak fractions corresponding to the hexamer form were collected and concentrated for cryoEM study.

For Nsp8, the cell pellets were resuspended with the buffer (20 mM Tris-HCl (pH 8.0), 500 mM NaCl, and 0.5 mM TCEP) containing RNase A (0.1 mg/ml, Qiagen), lysed by sonication, and cellular debris was removed by centrifugation. The supernatant containing the His_6-Nsp8 was loaded onto the Ni-NTA agarose column (Qiagen). The nickel column was extensively washed with wash buffer (20 mM Tris-HCl (pH 8.0), 500 mM NaCl, 20 mM imidazole, and 0.5 mM TCEP) and the protein was eluted with elution buffer (20 mM Tris-HCl (pH 8.0), 500 mM NaCl, 300 mM imidazole, and 0.5 mM TCEP). The eluted proteins were concentrated and subjected to Superdex 200 Increase 10/300 GL column (Cytiva) equilibrated with the buffer (20 mM Tris-HCl (pH 8.0), 250 mM NaCl, and 0.5 mM TCEP). The peak fractions were collected and concentrated for cryoEM study. Protein purity was assessed by SDS-PAGE at each purification step.

Negative-stain EM

5 µl of 0.02 mg/ml purified Nsp15 sample was applied onto glow-discharged ultrathin formvar/carbon supported copper 400-mesh grids (Electron Microscopy Sciences), blotted and stained with 2.0% uranyl acetate. Negative-stained grids were imaged on a Talos F200C transmission electron microscope (Thermo Fisher Scientific) operated at 200 kV.

CryoEM sample preparation and data acquisition

Three data sets were collected in separate TEM sessions. For reconstitution of Nsp15-RNA complex, pre-annealed dsRNA (chain 1: 5′-rUrCrUrUrArGrGrArGrArArUrGrArCrArArArArArArArArArArArArArArArArArArArA-3′, chain 2: 5′-rUrUrUrUrUrUrUrUrUrUrUrUrUrUrUrUrUrUrGrUrCrArUrUrCrUrCrCrUrArArGrA-3′) substrate was synthesized (Integrated DNA Technologies). For data set 1, Nsp15, Nsp8, and dsRNA were mixed by 1:0.5:1 molar ratio (7.5 µM Nsp15, 3.75 µM Nsp8, and 7.5 µM dsRNA) in a buffer (25 mM HEPES-NaOH, 150 mM NaCl, pH 7.5). For data sets 2 and 3, Nsp15, Nsp8, and dsRNA were mixed by 1:1:10 molar ratio (7.5 µM Nsp15, 7.5 µM Nsp8, and 75 µM dsRNA) in a buffer with lower salt (25 mM HEPES-NaOH, 100 mM NaCl, pH 7.5). The mixture was incubated on ice for 30-60 min before plunge-freezing. 4 ul aliquots of the mixture were applied to UltrAu foil R1.2/1.3 gold 300-mesh grids (Electron Microscopy Sciences). Grids were then blotted and vitrified in liquid ethane using Vitrobot Mark IV (Thermo Fisher Scientific). CryoEM data was collected in a Glacios (Thermo Fisher Scientific) equipped with Falcon-4 direct electron detector operated at 200 kV in electron counting mode. Movies were collected at a nominal magnification of 150,000× and a pixel size of 0.92 Å in EER format. A total dose of 52 $e^-/Å^2$ per movie was used with a dose rate of 5-6 $e^-/Å^2$/sec. 7,268, 10,001, and 5,150 movies were recorded for the data set 1, 2, and 3, respectively, by automated data acquisition with EPU.

CryoEM data processing

The movies from three data sets were imported into cryoSPARC software package (1) and subjected to patch motion correction and CTF estimation in cryoSPARC. For data set 1, reference-free manual particle picking in a small subset of data was performed to generate 2D templates for auto-picking. A total of 2,478,629 particles were picked initially, extracted, and down-sampled by a factor of 4, on which 2D classification was performed. 1,961,839 particles from 2D class averages were selected and re-extracted with full-resolution. 3D *ab initio* reconstruction was then performed to generate three initial volumes. A single dominant class containing 68% of the particles showed a feature of hexamer form Nsp15. Further classification did not yield any 3D classes containing RNA or Nsp8 density. For data sets 2 and 3, 2,983,555

and 1,730,135 particles were picked initially by using the templates generated from the data set 1, extracted, and down-sampled by a factor of 4, on which 2D classification was performed. Additional RNA densities were present in a subset of 2D classes, which are not present in the data set 1. 1,330,310 particles (data set 2) and 1,119,650 particles (data set 3) from 2D class averages were selected and re-extracted with full-resolution. 3D *ab initio* reconstruction was then performed to generate three initial volumes. A class containing 39% of the particles in each data set showed a clear feature of dsRNA density attached to the Nsp15 hexameric barrel. A class containing 37-38% of the particles in each data set showed a feature of hexamer form, which is similar to the class observed in the data set 1. The 3D classes representing hexamer form with no obvious RNA densities from the three data sets were combined and non-uniform refinement (2) was performed with D3 symmetry to yield the final 2.3 Å resolution map. The 3D classes representing RNA-bound form from the data sets 2 and 3 were combined and non-uniform refinement was performed with C1 symmetry to yield the final 2.7 Å resolution map. We noticed that RNA density in the 3D map was anisotropic, and the map could be an average of different conformational states. To further classify into possible different classes, heterogeneous refinement was performed to yield four classes. A class containing 24% of the particles showed strong RNA density along the top trimer (state 1) while two classes containing 53% of the particles showed strong RNA density along the bottom trimer (state 2). Each state was subjected to non-uniform refinement to yield the final 3.3 Å (state 1) and 3.1 Å (state 2) resolution maps, respectively. Any additional classification did not yield 3D classes with Nsp8, or Nsp15-RNA complex with more than one dsRNA bound to the Nsp15 hexamer. All resolution evaluation was performed based on the gold-standard criterion of Fourier shell correction (FSC) coefficient at 0.143 (3).

Model building and refinement

An atomic model derived from crystal structure of SARS-CoV-2 Nsp15 (PDB ID: 6VWW)(4) was docked into the cryoEM map of apo-Nsp15 using UCSF Chimera (5). The apo-Nsp15 model was refined with the phenix.real_space_refine module in Phenix, with secondary structure restraints and geometry restraints (6,7). The atomic models went through iterative cycles of manual adjustment in COOT (8) and real-space refinement in Phenix (9). For Nsp15-RNA complex consensus form, standard A-form double-stranded RNA was generated and docked together with apo-Nsp15 model into the cryoEM map using UCSF Chimera. The RNA model was manually adjusted while keeping proper RNA geometry using COOT. Nsp15-RNA complex states 1 and 2 models were built based on the consensus form by extending and refining the RNA strands. The final atomic models were validated using the comprehensive cryoEM validation tool implemented in Phenix (Table S1) (10). All structural figures were generated with UCSF ChimeraX (11).

Table S1. CryoEM data collection, refinement, and validation statistics

	Apo-Nsp15 (PDB: 8UD2) (EMDB: EMD-42144)	Nsp15-RNA consensus (PDB: 8UD3) (EMDB: EMD-42145)	Nsp15-RNA state 1 (PDB: 8UD4) (EMDB: EMD-42146)	Nsp15-RRNA state 2 (PDB: 8UD5) (EMDB: EMD-42147)
**Data collection**				
Magnification	150,000	150,000	150,000	150,000
Voltage (kV)	200	200	200	200
Electron exposure ($e^-/Å^2$)	52	52	52	52
Defocus range (μm)	-0.8 to -3.0	-0.8 to -3.0	-0.8 to -3.0	-0.8 to -3.0
Pixel size (Å)	0.92	0.92	0.92	0.92
Symmetry imposed	D3	C1	C1	C1
Initial particle images	7,192,319	4,713,690	4,713,690	4,713,690
Final particle images	2,294,976	961,569	227,075	511,627
Map resolution (Å)	2.33	2.67	3.25	3.13
FSC threshold	0.143	0.143	0.143	0.143
Map resolution range (Å)	2.0 - 2.5	2.2 - 4.0	2.4 - 5.0	2.3 - 5.0
**Refinement**				
Initial model used	PDB 6VWW	Apo-Nsp15	Nsp15-RNA consensus	Nsp15-RNA consensus
Model resolution (Å)	2.4	2.8	3.4	3.2
FSC threshold	0.5	0.5	0.5	0.5
Map sharpening B factor ($Å^2$)	112.1	104.3	99.5	117.9
No. non-hydrogen atoms	16530	17244	17498	17664
Protein residues	2076	2076	2076	2076
Nucleotides	0	34	46	54
B-factors				
Protein	41.65	47.82	60.80	40.61
Nucleotide	-	7.45	80.60	32.35
R.m.s. deviations				
Bond lengths (Å)	0.004	0.003	0.003	0.002
Bond angles (°)	0.953	0.523	0.562	0.490
Validation				
MolProbity score	1.37	1.41	1.42	1.21
Clash score	6.23	7.53	7.63	7.89
Poor rotamers (%)	1.08	0.65	0.43	0.27
Ramachandran plot				
Favored (%)	98.50	98.59	98.45	98.79
Allowed (%)	1.50	1.41	1.55	1.21
Disallowed (%)	0.00	0.00	0.00	0.00

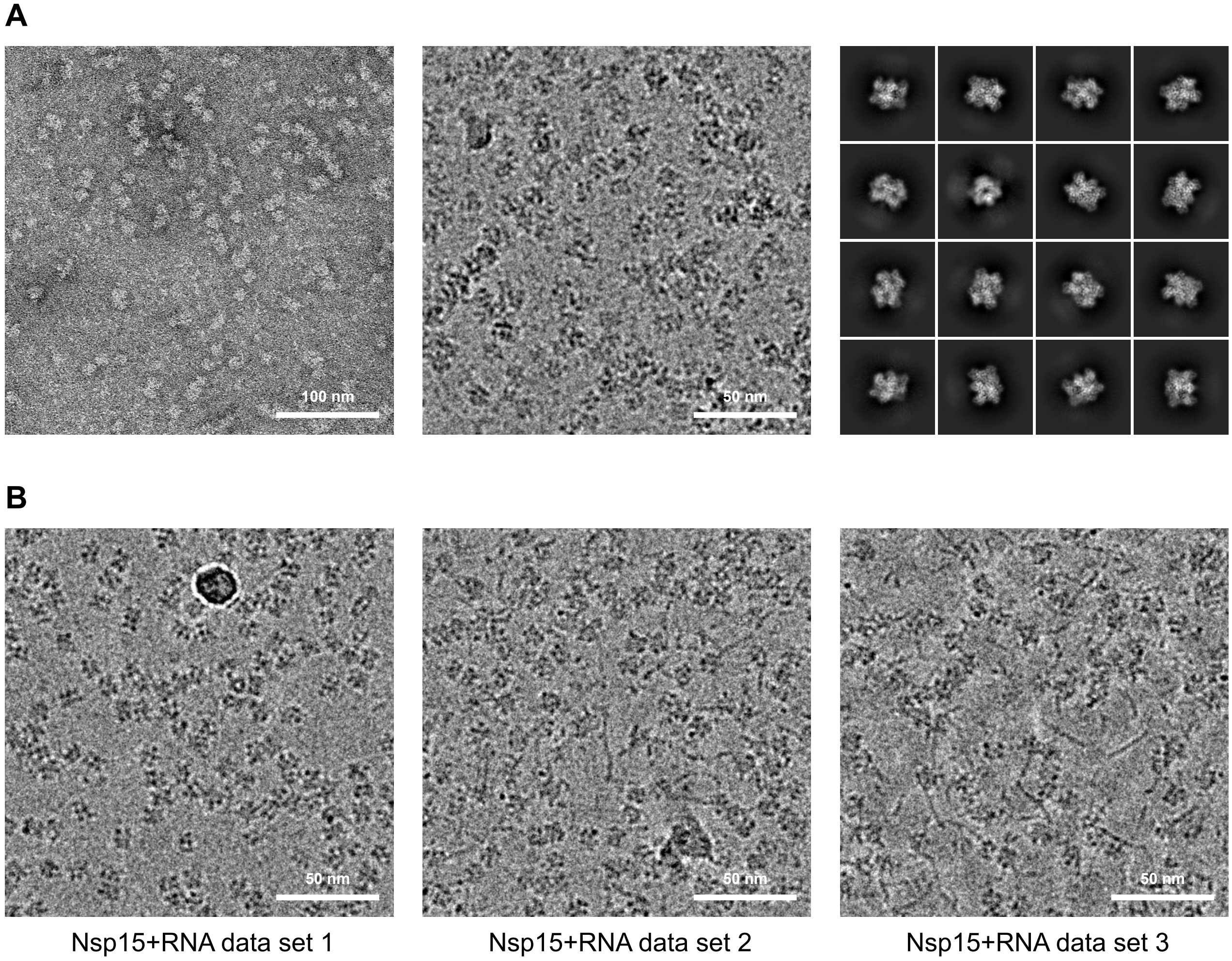

Fig. S1. Raw electron microscopy images of Nsp15. (A) Representative negative stain EM raw image (left), cryoEM raw image (middle), and cryoEM 2D class averages (right) of the apo-Nsp15. (B) Representative cryoEM raw images of Nsp15+RNA data sets.

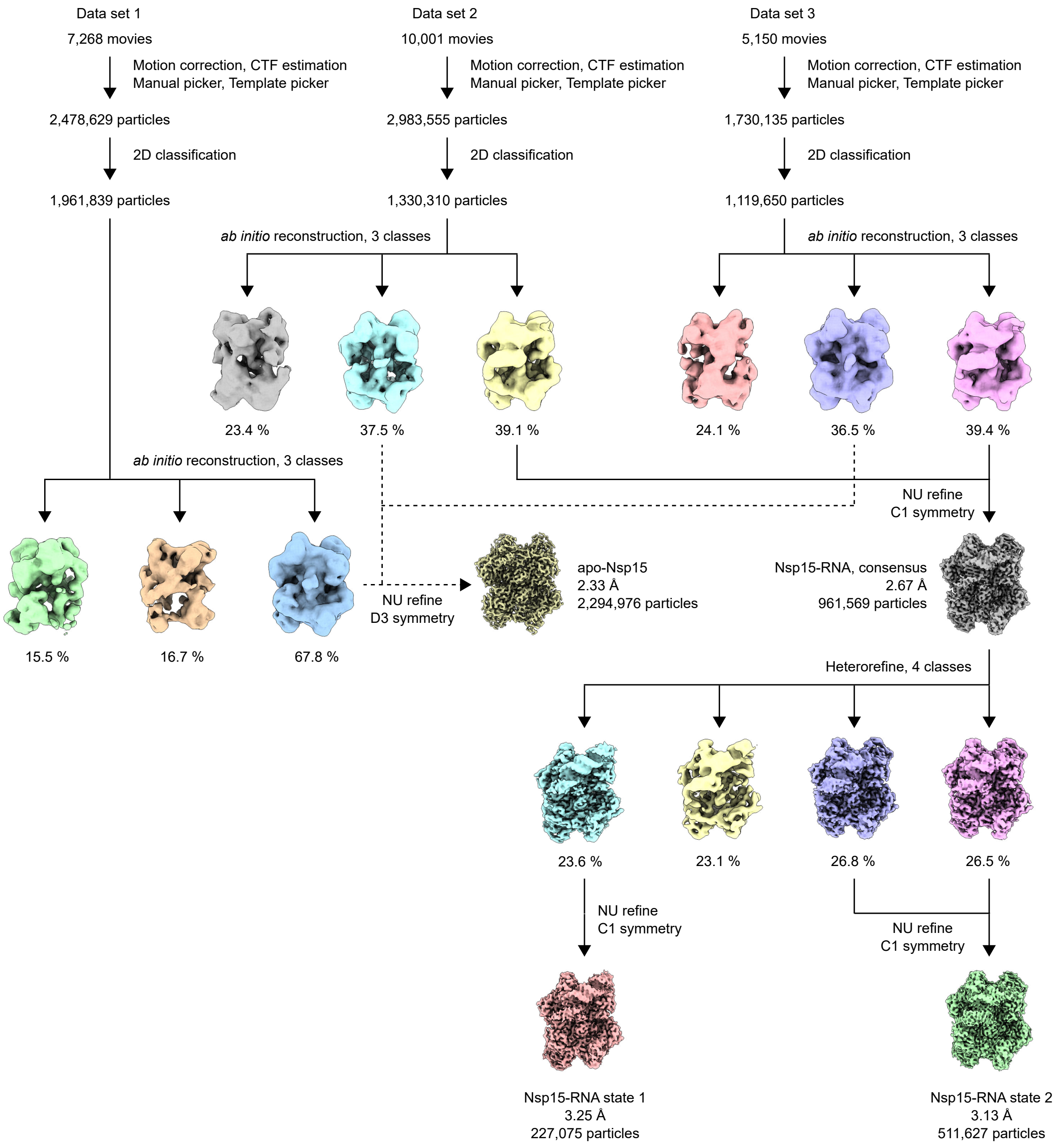

Fig. S2. Workflow and intermediate results of cryoEM image processing and 3D reconstruction of the apo-Nsp15 and the Nsp15-RNA complex.

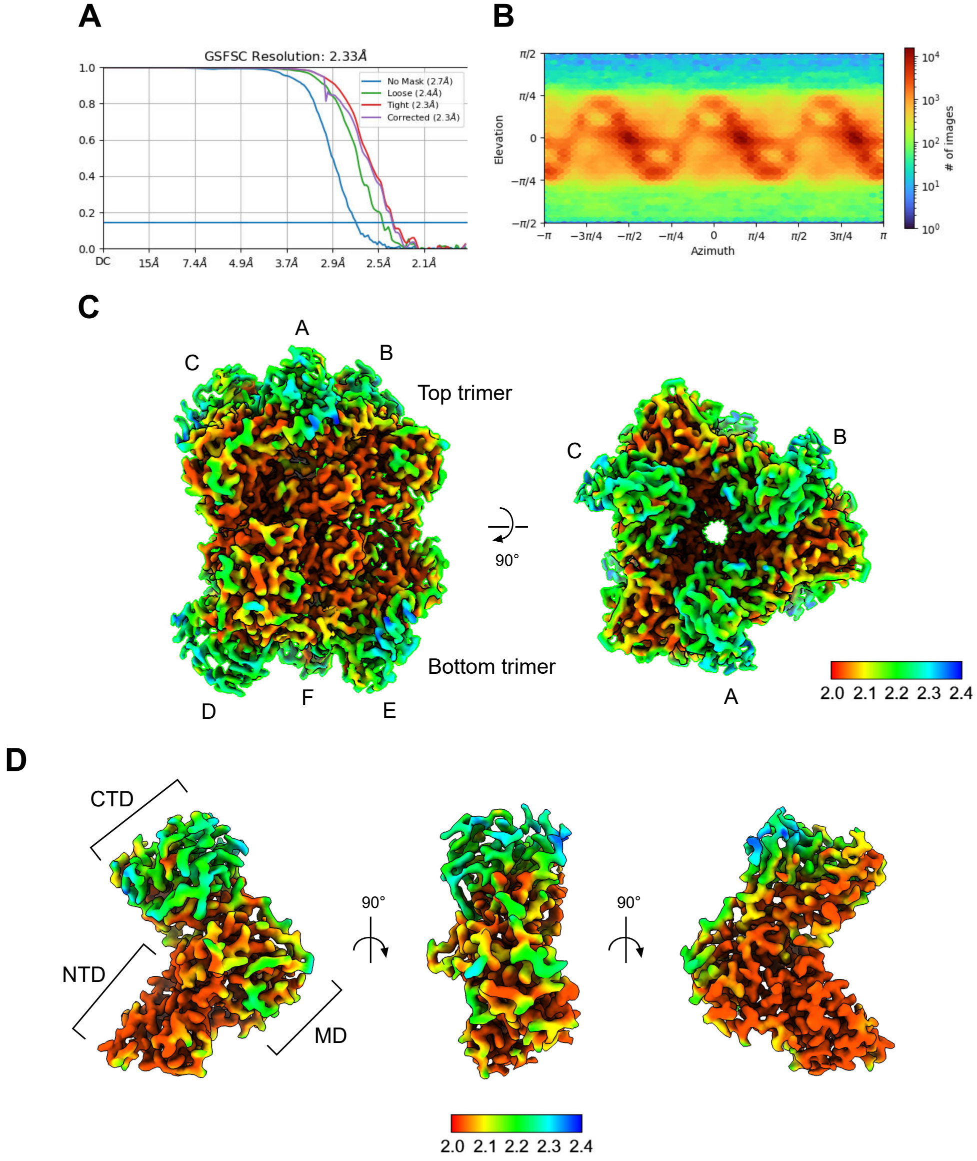

Fig. S3. Evaluation of the 3D reconstruction of apo-Nsp15. (A) Global resolution estimation of apo-Nsp15. (B) Angular distribution plot of the particles of apo-Nsp15. (C and D) Local resolution evaluation of the apo-Nsp15 hexamer (C) and one of its protomers (D). Resolution estimation is based on the gold standard Fourier shell correlation (FSC) coefficient of 0.143 criteria.

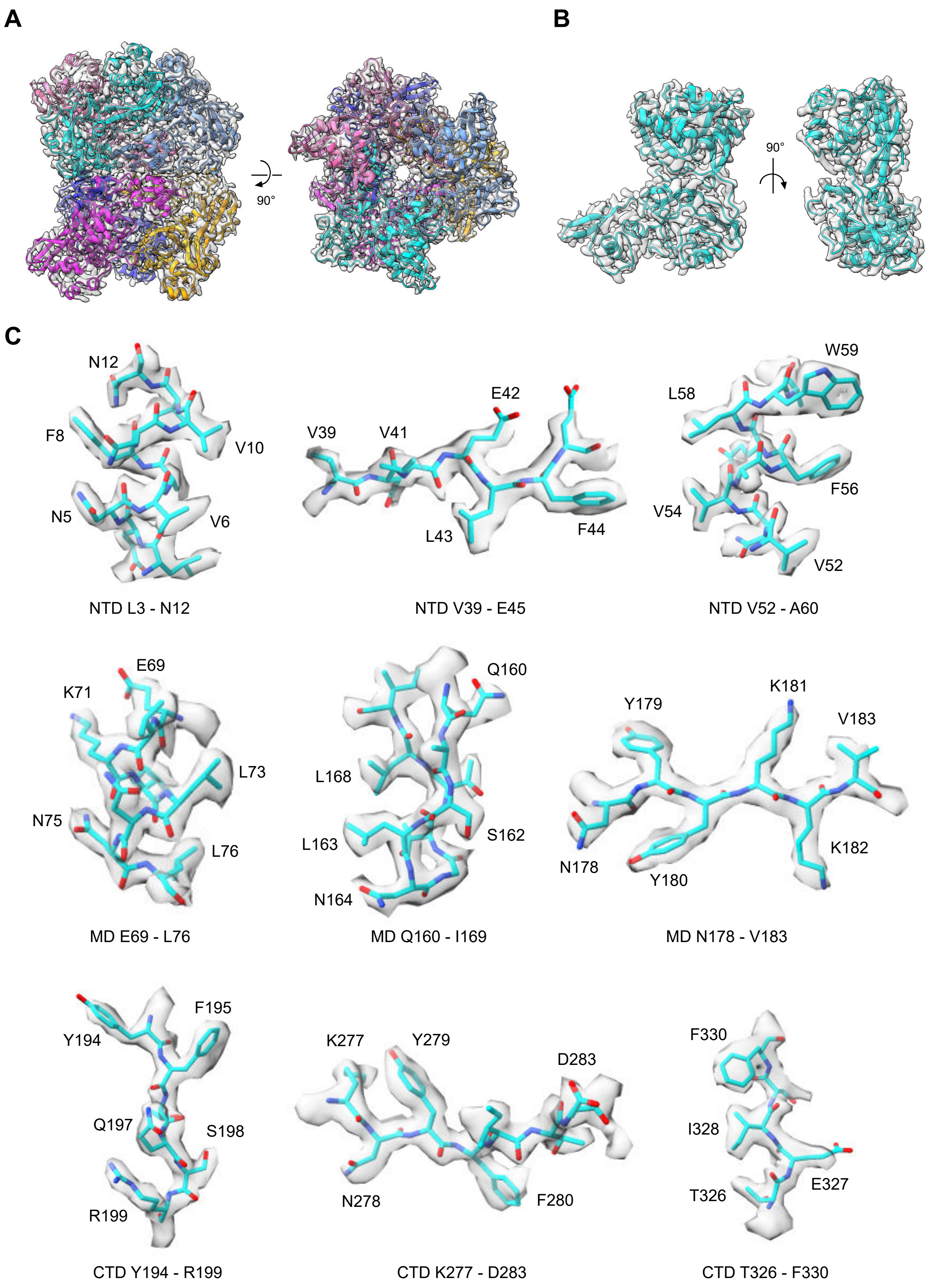

Fig. S4. Regions of the cryoEM density maps superposed with atomic models of the components of apo-Nsp15. Overall cryoEM density of apo-Nap15 hexamer (A), segmented cryoEM densities of representative protomer of apo-Nsp15 (B), and its local regions (C) are shown as semi-transparent surfaces superposed with atomic models of amino acid side chains (ribbons and sticks).

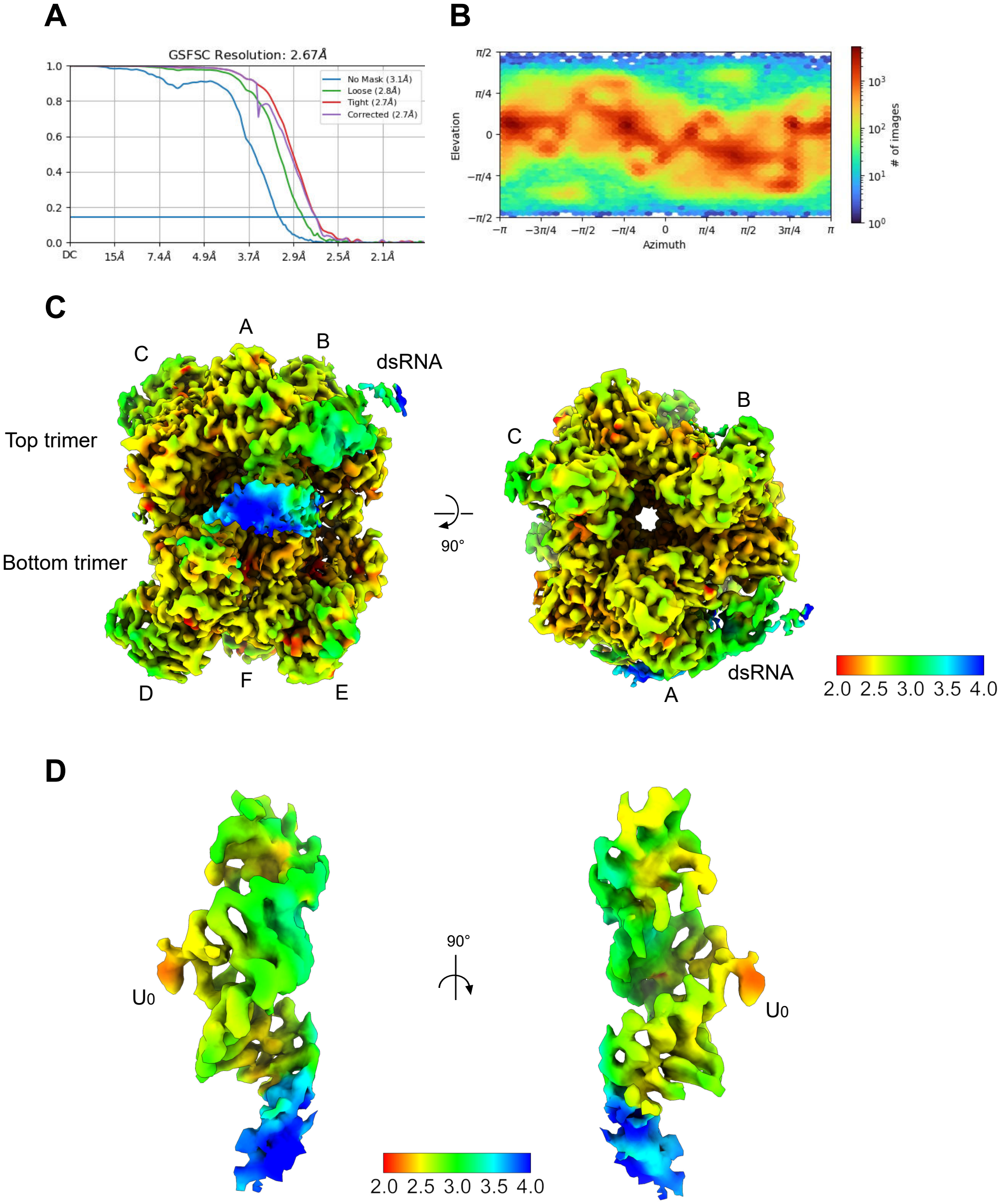

Fig. S5. Evaluation of the 3D reconstruction of Nsp15-RNA consensus structure. (A) Global resolution estimation of Nsp15-RNA. (B) Angular distribution plot of the particles of Nsp15-RNA. (C and D) Local resolution evaluation of the Nsp15-RNA (C) and its segmented RNA (D). Resolution estimation is based on the gold standard Fourier shell correlation (FSC) coefficient of 0.143 criteria.

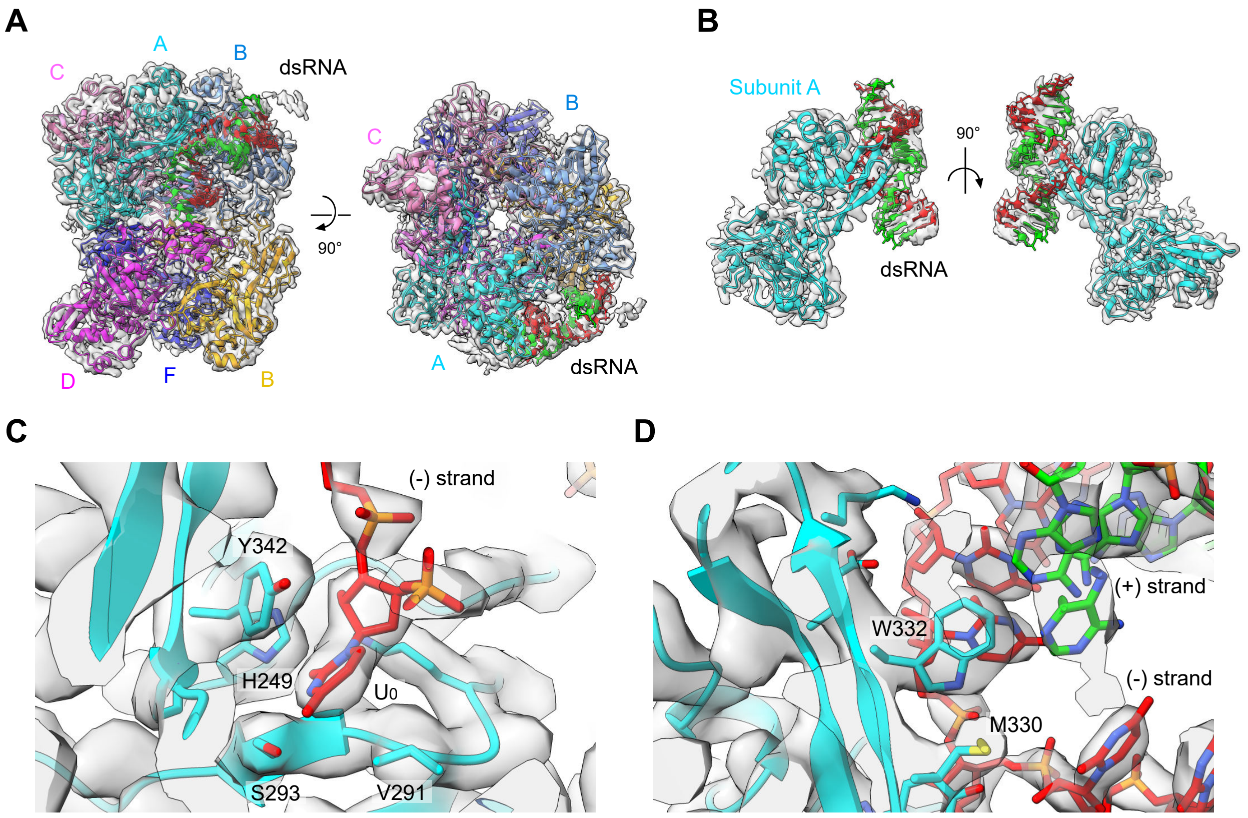

Fig. S6. Regions of the cryoEM density maps superposed with atomic models of the components of the Nsp15-RNA complex. Overall cryoEM density of Nap15-RNA (A), segmented cryoEM densities of the protomer A with the bound RNA (B), a local region around the U_0 base (C), and a local region around the base-flipping residues W332 and M330 (D) are shown as semi-transparent surfaces superposed with atomic models of amino acid side chains (ribbons and sticks).

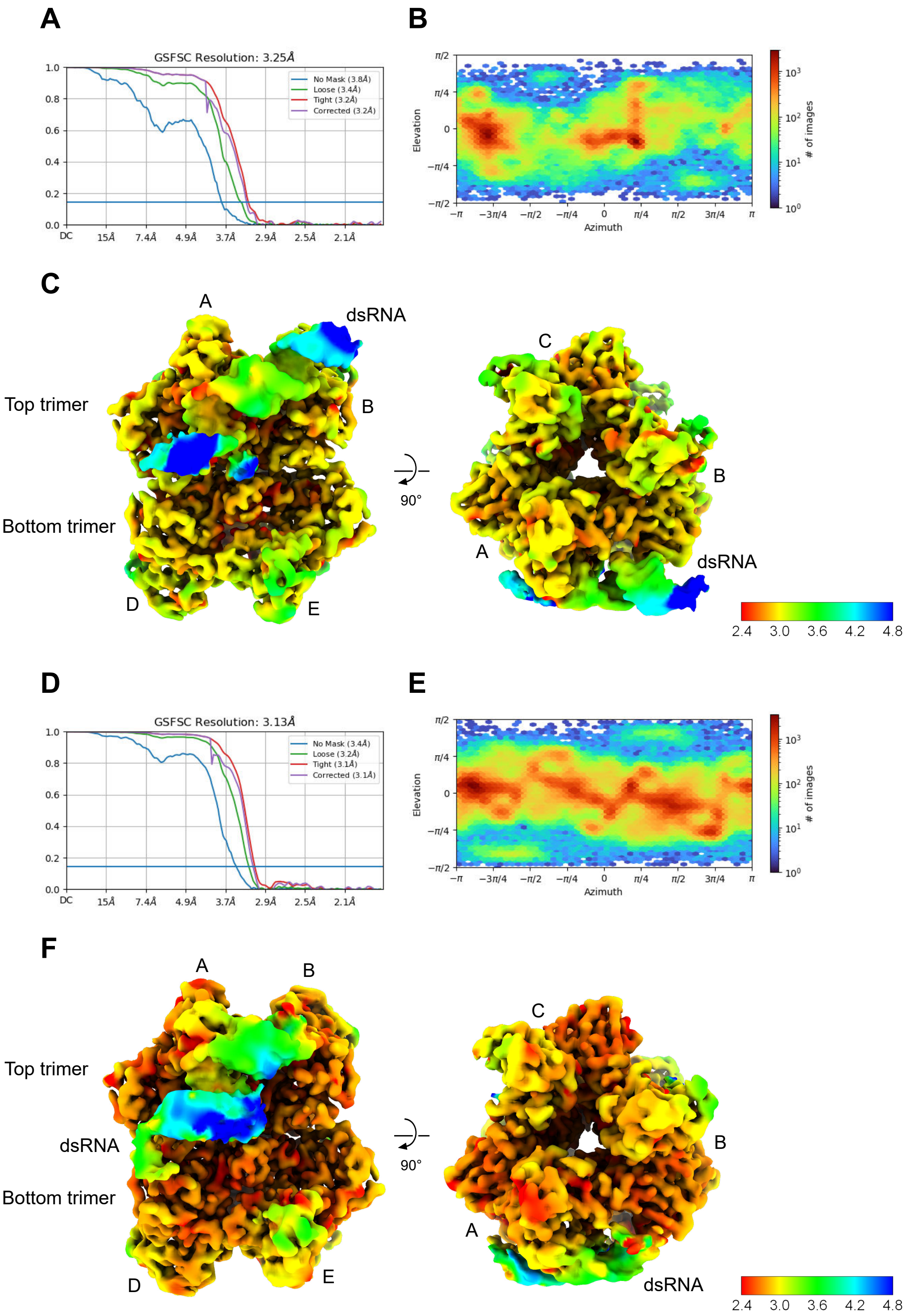

Fig. S7. Evaluation of the 3D reconstruction of two states of the Nsp15-RNA complex. (A and D) Global resolution estimation of Nsp15-RNA state 1 (A) and state 2 (D). (B and E) Angular distribution plot of the particles of Nsp15-RNA state 1 (B) and state 2 (E). (C and F) Local resolution evaluation of the Nsp15-RNA state 1 (C) and state 2 (F). Resolution estimation is based on the gold standard Fourier shell correlation (FSC) coefficient of 0.143 criteria.

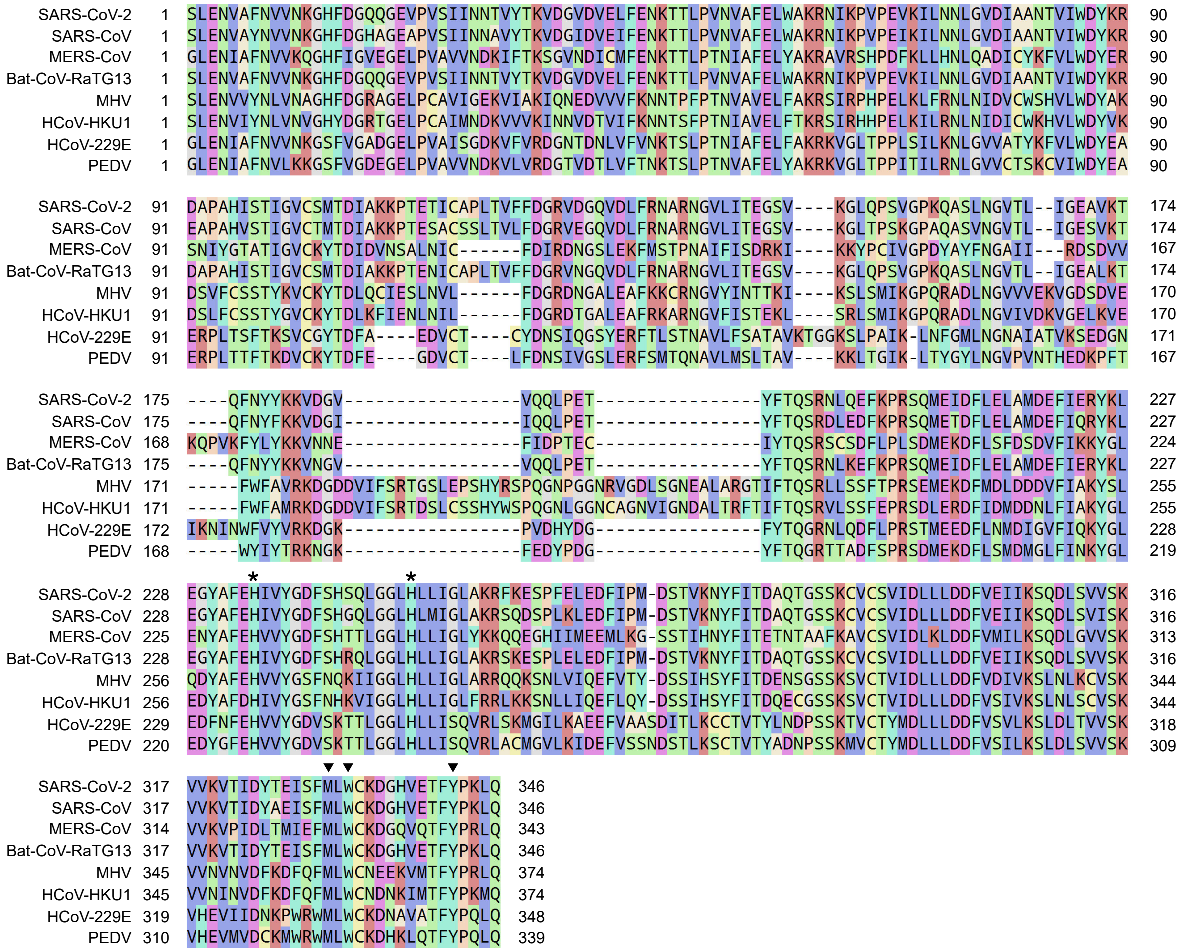

Fig. S8. Sequence alignment of coronavirus Nsp15. The functionally important residues in SARS-CoV-2 Nsp15 are marked for comparison with other coronavirus Nsp15s. Amino acid residues responsible for base-flipping are marked with triangles. Active site histidine residues are marked with asterisks. MERS-CoV, Middle East respiratory syndrome coronavirus; MHV, mouse hepatitis virus; PEDV, porcine epidemic diarrhea virus.

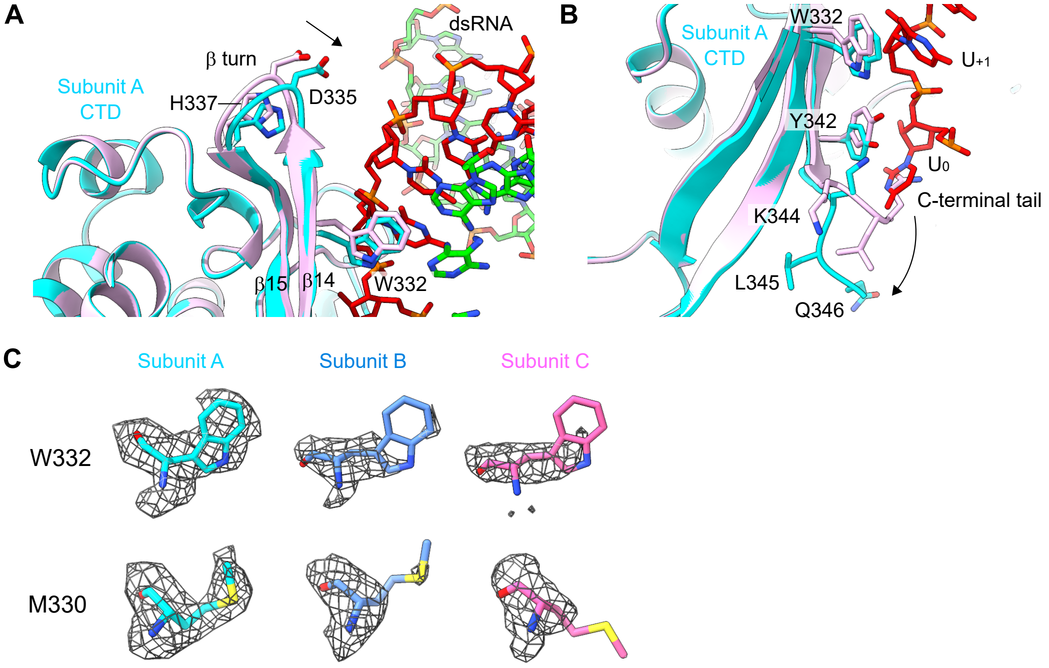

Fig. S9. Structural remodeling of Nsp15 upon RNA binding. (A and B) The structural superimposition of the subunit A of the RNA-bound form (ribbon model in cyan) and apo form (ribbon model in light purple). The fine-adjustment of the position of β14-tun-β15 region of subunit A's CTD (A) and the structural remodeling of the C-terminal tail of subunit A (B) were observed. C-terminal residues - ^{344}KLQ^{346} swings away from the active site center upon RNA binding. The consensus form of the Nsp15-RNA structure is used for comparison. (C) The comparison of the density of W332 and M330 in different subunits at the same iso-surface threshold level (threshold: 0.32). The densities of subunit A show the clearer side-chain features of both W332 and M330 than those of the other subunits.

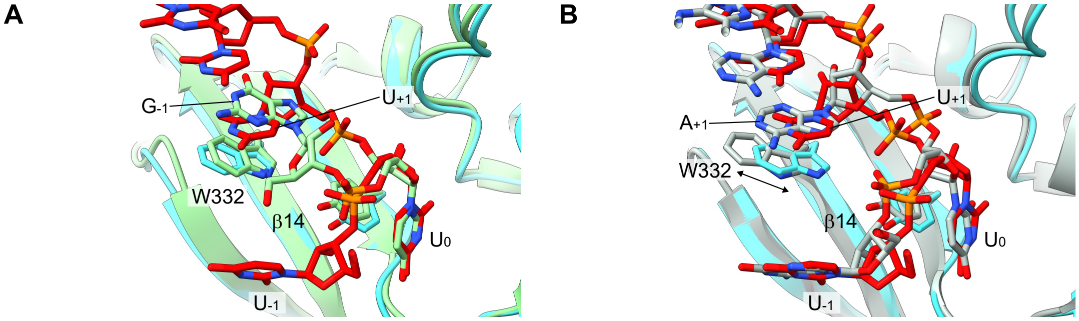

Fig. S10. Comparison of SARS-CoV-2 Nsp15-RNA structures. Close-up views of the superimposition of the Nsp15-RNA consensus structure from this study (cyan) with short ssDNA-bound crystal structure (PDB ID: 6X1B, light green)(A) and with 52-bp dsDNA-bound cryoEM structure (PDB ID: 7TJ2, light gray)(B).

References

1. Punjani, A., Rubinstein, J.L., Fleet, D.J. and Brubaker, M.A. (2017) cryoSPARC: algorithms for rapid unsupervised cryo-EM structure determination. *Nat Methods*, **14**, 290-296.
2. Punjani, A., Zhang, H. and Fleet, D.J. (2020) Non-uniform refinement: adaptive regularization improves single-particle cryo-EM reconstruction. *Nat Methods*, **17**, 1214-1221.
3. Chen, S., McMullan, G., Faruqi, A.R., Murshudov, G.N., Short, J.M., Scheres, S.H. and Henderson, R. (2013) High-resolution noise substitution to measure overfitting and validate resolution in 3D structure determination by single particle electron cryomicroscopy. *Ultramicroscopy*, **135**, 24-35.
4. Kim, Y., Jedrzejczak, R., Maltseva, N.I., Wilamowski, M., Endres, M., Godzik, A., Michalska, K. and Joachimiak, A. (2020) Crystal structure of Nsp15 endoribonuclease NendoU from SARS-CoV-2. *Protein Sci*, **29**, 1596-1605.
5. Pettersen, E.F., Goddard, T.D., Huang, C.C., Couch, G.S., Greenblatt, D.M., Meng, E.C. and Ferrin, T.E. (2004) UCSF Chimera--a visualization system for exploratory research and analysis. *J Comput Chem*, **25**, 1605-1612.
6. Adams, P.D., Afonine, P.V., Bunkoczi, G., Chen, V.B., Davis, I.W., Echols, N., Headd, J.J., Hung, L.W., Kapral, G.J., Grosse-Kunstleve, R.W. *et al.* (2010) PHENIX: a comprehensive Python-based system for macromolecular structure solution. *Acta Crystallogr D Biol Crystallogr*, **66**, 213-221.
7. Afonine, P.V., Grosse-Kunstleve, R.W., Echols, N., Headd, J.J., Moriarty, N.W., Mustyakimov, M., Terwilliger, T.C., Urzhumtsev, A., Zwart, P.H. and Adams, P.D. (2012) Towards automated crystallographic structure refinement with phenix.refine. *Acta Crystallogr D Biol Crystallogr*, **68**, 352-367.
8. Emsley, P., Lohkamp, B., Scott, W.G. and Cowtan, K. (2010) Features and development of Coot. *Acta Crystallogr D Biol Crystallogr*, **66**, 486-501.
9. Afonine, P.V., Poon, B.K., Read, R.J., Sobolev, O.V., Terwilliger, T.C., Urzhumtsev, A. and Adams, P.D. (2018) Real-space refinement in PHENIX for cryo-EM and crystallography. *Acta Crystallogr D Struct Biol*, **74**, 531-544.
10. Afonine, P.V., Klaholz, B.P., Moriarty, N.W., Poon, B.K., Sobolev, O.V., Terwilliger, T.C., Adams, P.D. and Urzhumtsev, A. (2018) New tools for the analysis and validation of cryo-EM maps and atomic models. *Acta Crystallogr D Struct Biol*, **74**, 814-840.
11. Goddard, T.D., Huang, C.C., Meng, E.C., Pettersen, E.F., Couch, G.S., Morris, J.H. and Ferrin, T.E. (2018) UCSF ChimeraX: Meeting modern challenges in visualization and analysis. *Protein Sci*, **27**, 14-25.
